# Proline transporters ProT and PutP are required for *Staphylococcus aureus* infection

**DOI:** 10.1371/journal.ppat.1011098

**Published:** 2023-01-18

**Authors:** McKenzie K. Lehman, Natalie A. Sturd, Fareha Razvi, Dianne L. Wellems, Steven D. Carson, Paul D. Fey

**Affiliations:** University of Nebraska Medical Center, Department of Pathology and Microbiology, Omaha, Nebraska, United States of America; Columbia University, UNITED STATES

## Abstract

Proline acquired via specific transporters can serve as a proteinogenic substrate, carbon and nitrogen source, or osmolyte. Previous reports have documented that *Staphylococcus aureus*, a major community and nosocomial pathogen, encodes at least four proline transporters, PutP, OpuC, OpuD, and ProP. A combination of genetic approaches and ^3^H-proline transport assays reveal that a previously unrecognized transporter, ProT, in addition to PutP, are the major proline transporters in *S*. *aureus*. Complementation experiments using constitutively expressed non-cognate promoters found that proline transport via OpuD, OpuC, and ProP is minimal. Both proline biosynthesis from arginine and proline transport via ProT are critical for growth when *S*. *aureus* is grown under conditions of high salinity. Further, proline transport mediated by ProT or PutP are required for growth in media with and without glucose, indicating both transporters function to acquire proline for proteinogenic purposes in addition to acquisition of proline as a carbon/nitrogen source. Lastly, inactivation of *proT* and *putP* resulted in a significant reduction (5 log_10_) of bacterial burden in murine skin-and-soft tissue infection and bacteremia models, suggesting that proline transport is required to establish a *S*. *aureus* infection.

## Author summary

The amino acid proline has multiple functions in the cell, including use as a basic building block for protein synthesis, carbon and nitrogen source, and compatible solute that protects the cells from osmotic stress. These studies reveal that the pathogen *Staphylococcus aureus* encodes two primary proline transporters, PutP and the newly described ProT, that are required for supporting growth in skin and soft tissue infections. Proline transport by either PutP or ProT is sufficient to support maximal growth when proline is utilized as a key carbon and nitrogen source, however, only ProT is required for growth in high salt conditions.

## Introduction

*Staphylococcus aureus* is the leading cause of skin and soft tissue infections [1]. Glucose catabolism is required for establishment of *S*. *aureus*-mediated skin and soft tissue infections [2], therefore, we hypothesize that pathways repressed via carbon catabolite repression (CCR) mediated by CcpA, including those that synthesize proline, are not essential for establishment of an infection [3–5]. These observations suggest that the proline required for growth during the initial phases of skin abscess formation must be acquired from the host milieu and imported via proline transporters or acquired through peptide transport [6].

Although glycolysis, and therefore glucose catabolism, is required for the establishment of a *S*. *aureus* skin abscess [2], glucose becomes depleted from the abscess over time due to lack of vascularization [2,7–10]. Once glucose is depleted, *S*. *aureus* must rely on catabolism of secondary carbon sources (i.e., amino acids and peptides) and gluconeogenesis for proliferation in the wound [6,11]. Glutamate, and amino acids that serve as glutamate precursors, including proline, arginine, and histidine, are the primary carbon sources within this niche [11]. Indeed, proline is rapidly consumed from media lacking glucose, suggesting that transport and catabolism of proline is important for *S*. *aureus* proliferation when glucose is absent [11].

In addition to its function as a proteinogenic amino acid and carbon source, proline also serves as a critical osmolyte that protects against osmotic stress [12–17]. Current literature suggests that *S*. *aureus* encodes four proline transporters, PutP, OpuD, ProP, and OpuC [17–26]. With this knowledge, we sought to document the importance of each proline transporter during *S*. *aureus* growth in glucose-rich and -limited conditions, in addition to growth conditions with elevated NaCl levels. Utilizing growth analysis in chemically defined media (CDM) and ^3^H-proline transport assays, we identified a previously uncharacterized proline transporter, ProT. Furthermore, utilizing murine models of skin and soft tissue infection and bacteremia, we determined that proline transport is required to establish *a S*. *aureus* infection.

## Results

### Selection of isolates resistant to the toxic proline analogue DHP

**Fig 1:**
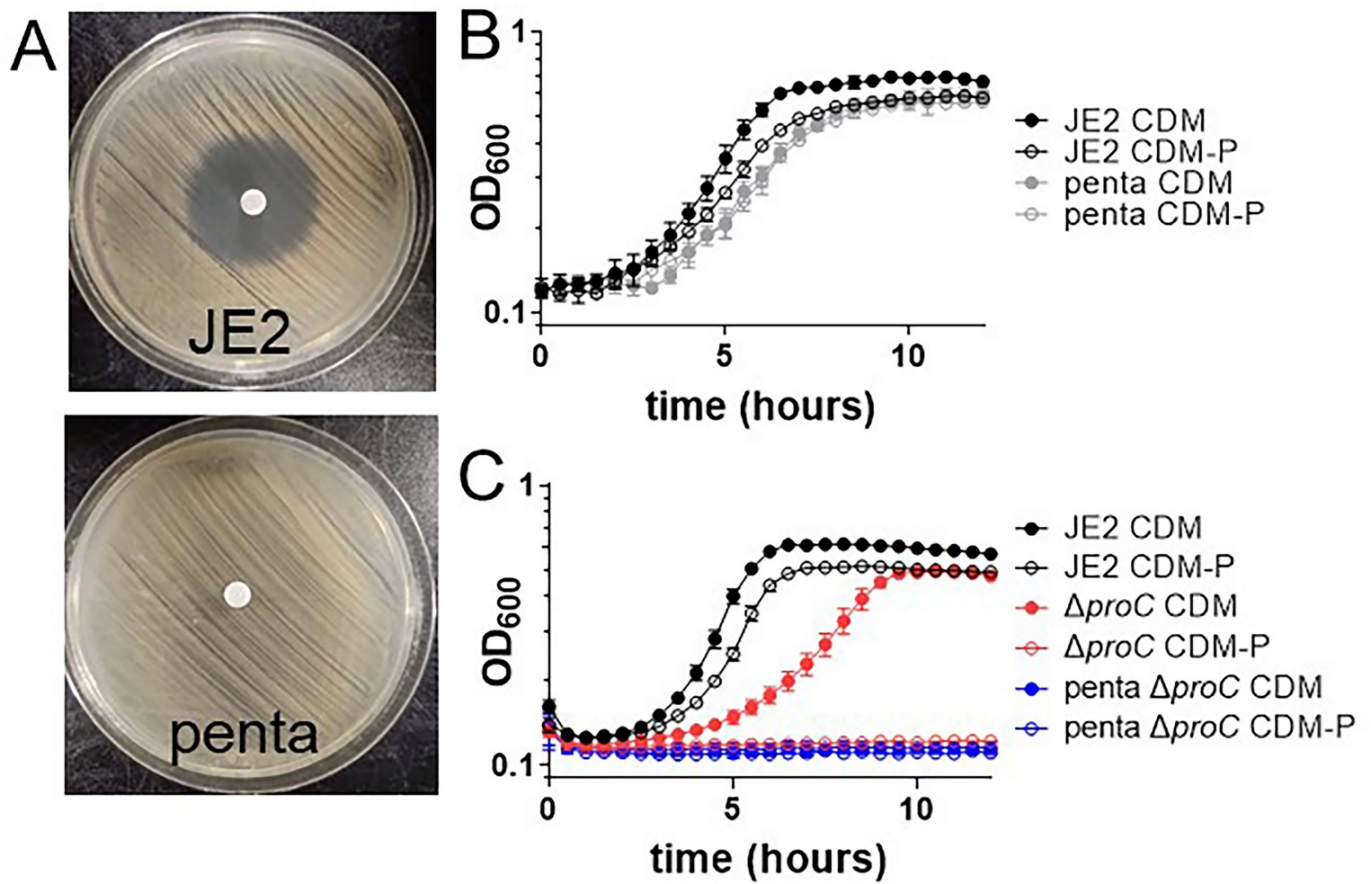
B7H15_03660 encodes an additional proline transporter. A) A large zone of inhibition surrounds the disc containing the toxic proline analog 3,4 dehydro-DL-proline (DHP). When all five putative transporters, including the newly identified B7H15_03660, are inactivated, the penta mutant is no longer susceptible to DHP. B) Growth analysis of JE2 and the penta mutant in CDM and CDM-P. C) Growth analysis of JE2, Δ*proC*, and penta Δ*proC* in CDM and CDM-P show an inability of the penta Δ*proC* mutant to grow in CDM. Data (B and C) are represented by the mean ± SD (n = 3).

Early studies of proline transport in *S*. *aureus* indicated the presence of both high- and low-affinity transporter(s) [17,19]. Follow-up studies determined that *S*. *aureus* encoded orthologs to the low-affinity transporters OpuD and ProP characterized in *Bacillus subtilis* and *Escherichia coli*, respectively [21,24]. *S*. *aureus* also encodes an ortholog to the *E*. *coli* high-affinity transporter, PutP [22,23,25,26]. Further, it has been suggested that OpuC may also function as a proline transporter [21]. To determine if proline transport in *S*. *aureus* is inclusive of these four transporters, a JE2 Δ*opuC* Δ*opuD* Δ*proP* Δ*putP* mutant was constructed and tested for susceptibility to the toxic proline analog 3,4 dehydro-DL-proline (DHP) (S1A Fig). Disk-diffusion studies revealed that JE2 Δ*opuC* Δ*opuD* Δ*proP* Δ*putP* was still susceptible to DHP, documenting that DHP could still be transported. However, resistant colonies noted within the zone of inhibition were isolated and found to be completely resistant to DHP (S1A Fig). Amino acid consumption assays of four DHP-resistant isolates (DHP 1–4) determined that, in contrast to JE2 WT and JE2 Δ*opuC* Δ*opuD* Δ*proP* Δ*putP*, DHP 2–4 did not consume exogenous proline from the medium, and DHP-1 only consumed ~50% of added proline, suggesting that DHP 1–4 may have mutations in an unknown proline transporter (S1B Fig). Whole-genome sequencing revealed that DHP 1–4 had SNPs (three resulting in frameshifts; Table 1) disrupting B7H15_03660 (*S*. *aureus* JE2; NCBI Reference Sequence: NZ_CP020619.1) encoding a predicted transmembrane protein. To test the hypothesis that B7H15_03660 encodes a proline transporter, an allelic replacement mutant was constructed and introduced into JE2 Δ*opuC* Δ*opuD* Δ*proP* Δ*putP* via ϕ11 transduction. The resulting strain JE2 Δ*opuC* Δ*opuD* Δ*proP* Δ*putP* Δ*B7H15_03660* (hereafter referred to as the penta mutant) was resistant to DHP, similar to isolates DHP 1–4 (Figs 1A and S1A). Additionally, the penta mutant had reduced growth yield (reaching a lower final OD_600_) in CDM, phenocopying the growth yield observed when JE2 is grown in CDM lacking proline (CDM-P) (Fig 1B). These data reveal that exogenous proline is not utilized as a carbon source in the penta mutant even if proline is added to the medium. To confirm that the growth observed in the penta mutant was dependent on proline biosynthesis, we transduced a *proC* mutation into the penta mutant. *proC* encodes pyrroline-5-carboxylate reductase and is the terminal enzyme responsible for proline synthesis (S1C Fig). Therefore, JE2 Δ*proC* is unable to grow in CDM-P due to a lack of exogenous proline and an inability to synthesize proline using arginine as a substrate [5]. The penta Δ*proC* strain was unable to grow in CDM or CDM-P (Fig 1C), indicating that the penta mutant, in contrast to JE2 Δ*opuC* Δ*opuD* Δ*proP* Δ*putP*, did not have a functional proline transporter. Overall, these data show that *S*. *aureus* may encode up to five proline transporters, including B7H15_03660.

**Table 1:** Whole genome sequencing of DHP resistant strains.

strain	amino acid change	locus	protein encoded
DHP-1	gly➔ser (120)	B7H15_03660	transmembrane protein
DHP-2	frameshift (350)	B7H15_03660	transmembrane protein
DHP-3	trp➔stop (38)	B7H15_03660	transmembrane protein
DHP-4	frameshift resulting in premature stop (57)	B7H15_03660	transmembrane protein

### B7H15_03660 encodes a functional proline transporter, ProT

We next sought to determine which of the five putative proline transporters serve as functional proline transporters. An initial growth screen was performed in CDM and CDM-P with strains where individual proline transporters were inactivated in JE2 (S2 Fig). Although extended lag phases were observed, a mutation in any one of the proposed proline transporters did not result in reduced growth yield relative to that observed in the penta mutant, suggesting that there are at least two functional proline transporters. Moreover, supporting this conclusion, resistant colonies were observed with JE2 Δ*opuC* Δ*opuD* Δ*proP* Δ*putP* (S1A Fig), but not JE2 WT (Fig 1A), indicating that there were multiple proline transporters rendering the cells susceptible to the toxic proline analogue.

**Fig 2:**
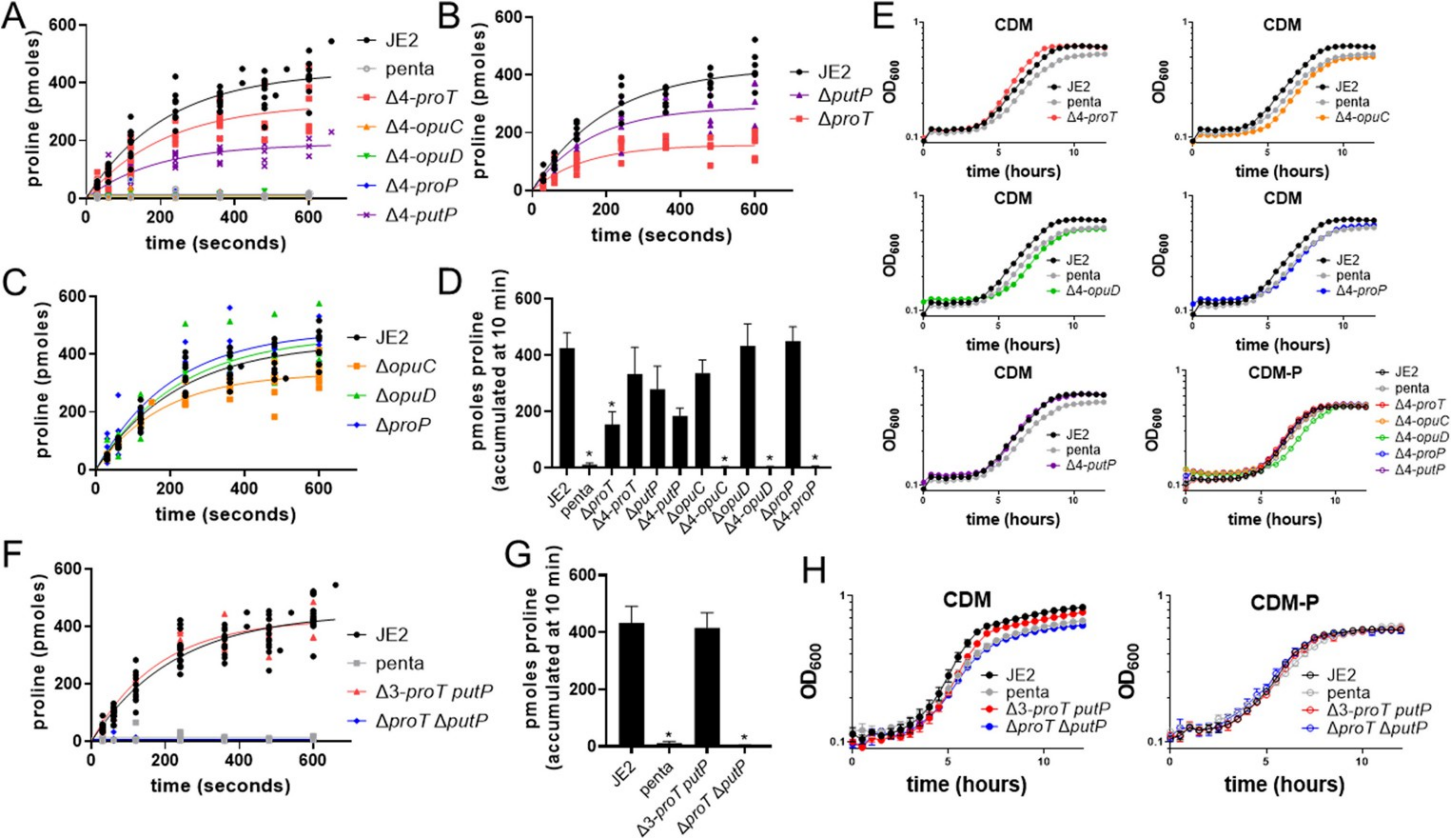
B7H15_03660 encodes a novel proline transporter, ProT. ^3^H-proline transport assays of various proline transporter mutants including A) single transporter expression strains in which single transporters were expressed from their native loci. These strains are labeled as Δ4-proline transporter still intact (i.e., Δ4-*proT* is genetically Δ*opuC* Δ*opuD* Δ*proP* Δ*putP*). Additional ^3^H-proline transport assays of single deletion strains including B) Δ*proT* and Δ*putP* and C) Δ*opuD*, Δ*opuC*, and Δ*proP* were performed. D) Measured amounts of accumulated proline after 10 minutes of the proline transporter strains with a single transporter intact (i.e., Δ4-*proT*) or the single transporter mutated (i.e., Δ*proT*) were determined. E) Growth analysis of strains encoding single transporters in CDM and CDM-P confirmed ProT and PutP support maximal growth yield in CDM. F) ^3^H-proline transport assays, G) measured amounts of accumulated proline, and H) growth analysis in CDM and CDM-P of the Δ*proT* Δ*putP* and Δ3-*proT putP* (genotypically Δ*opuC* Δ*opuD* Δ*proP*) strains confirm ProT and PutP are the two proline transporters in *S*. *aureus*. All ^3^H-proline transport assay data (A, B, C, F) are standardized to JE2 to account for experimental variations and fit by nonlinear regression using a first-order equation of technical duplicates ran three independent times. Accumulated ^3^H-proline data (D and G) are represented by the mean ± SD (n = 5–6). Kruskal-Wallis multiple comparisons identified statistically significant differences (* p<0.05). Growth curve data (E and H) are represented by the mean ± SD (n = 3).

Next, JE2 strains were generated in which combinations of four of the five putative transporter loci were inactivated, resulting in strains where each of the different putative transporters was individually expressed from its native locus and promoter. ^3^H-proline transport assays were performed to assess the function of each proposed proline transporter. In this assay, ^3^H-proline transport was measured over a 10-minute time course using JE2 as control (Fig 2A and 2D). These assays revealed that the newly identified transporter encoded by B7H15_03660 was a functional proline transporter, supporting accumulation of approximately 75% the amount of proline accumulated by JE2 at 10 minutes (Fig 2D). This newly identified transporter was named ProT, for proline transporter. Sequence analysis revealed that ProT is highly conserved in *S*. *aureus* with protein sequence homology in all sequenced *S*. *aureus* strains ranging from 97–100%. Moreover, among other staphylococcal species, this protein retains high sequence conservation with >70% homology, suggesting that ProT is highly conserved among other staphylococcal species (S3 Fig) [27].

Analysis of the other single-expression strains revealed that PutP is the only other functional proline transporter, enabling accumulation of approximately 35% of the proline amassed by JE2 over 10 minutes (Fig 2A and 2D). Transport assays using strains with only one transporter inactivated further confirmed that PutP and ProT are the primary proline transporters under the conditions used in this experiment. The Δ*proT* mutant phenocopied the transport of the single expression *putP* strain (Δ4-*putP)*, and the Δ*putP* mutant phenocopied the single expression *proT* strain (Δ4-*proT*) (Fig 2B and 2D). Growth analyses of Δ4-*putP* and Δ4-*proT* in CDM and CDM-P further confirm that only ProT and PutP function to adequately transport proline, as either is necessary and sufficient to phenocopy growth of JE2 in CDM (Fig 2E). Strains expressing OpuC, OpuD, or ProP individually do not phenocopy growth of JE2 in CDM, but instead phenocopy growth observed in the penta mutant, suggesting they are not transporting proline (Fig 2E). There was a slight reduction in proline accumulation in the Δ*opuC* mutant (Fig 2C and 2D), suggesting that OpuC may contribute to proline accumulation, albeit likely indirectly, as when OpuC was expressed without other known transporters present (Δ4-*opuC*), proline accumulation was not observed (Fig 2A and 2D). JE2 Δ*proT* Δ*putP* phenocopied the penta mutant in proline transport assays (Fig 2F and 2G) and growth in CDM (Fig 2H), whereas the Δ3-*proT putP* strain (genotypically Δ*opuC* Δ*opuD* Δ*proP*) transported proline at similar rates and grew to the same stationary phase cell density in CDM as JE2 (Fig 2F–2H). In summation, these data show that ProT and PutP are the two primary proline transporters in *S*. *aureus*.

### OpuD, OpuC, and ProP are not primary proline transporters in *S*. *aureus*

**Fig 3:**
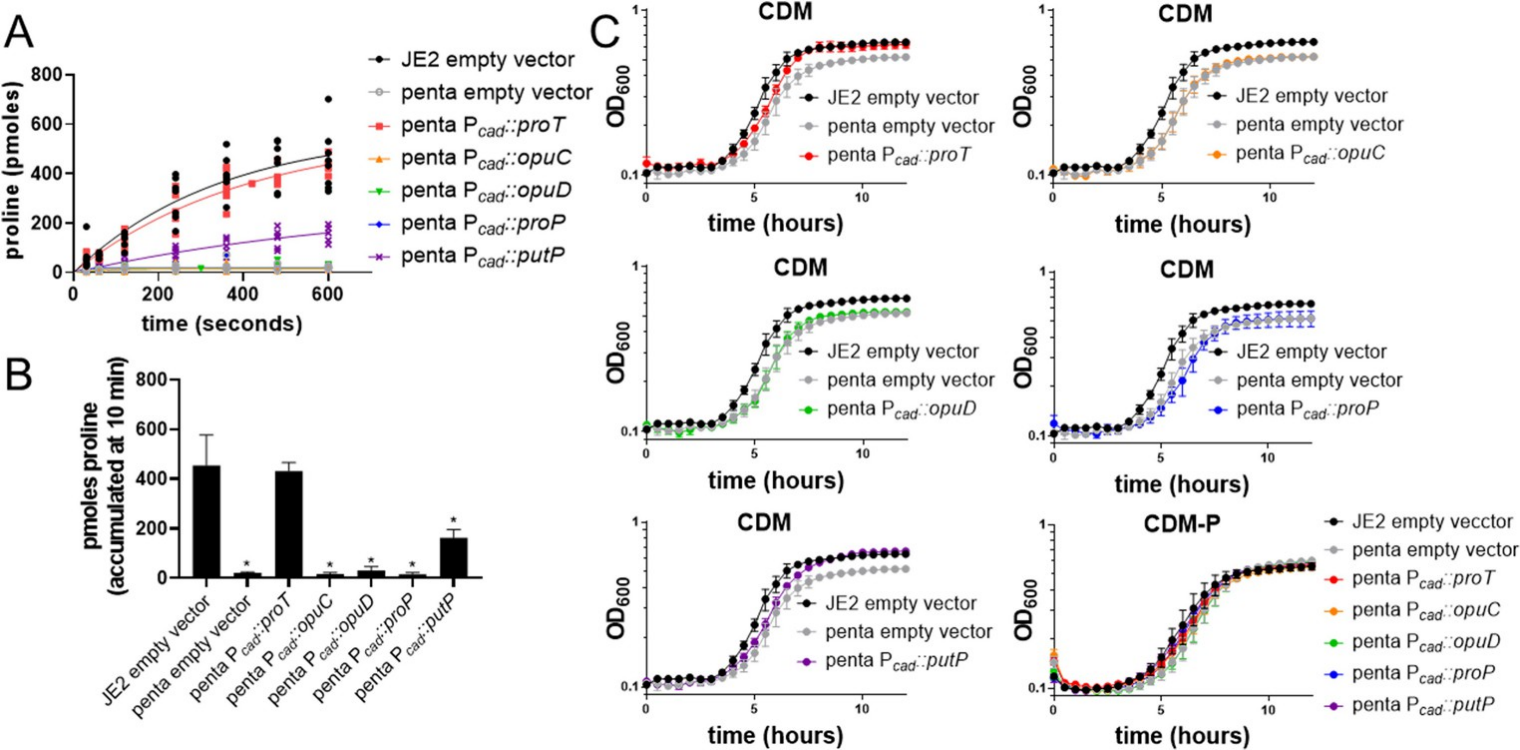
Proline is not transported by OpuC, OpuD, or ProP. A) ^3^H-proline transport assays, B) measured amounts of proline accumulated at 10 minutes, and C) growth analysis in CDM and CDM-P of JE2 and penta pBK123 (empty vector), pML1 (P_cad_::*proT*), pML2 (P_cad_::*opuC*), pML3 (P_cad_::*opuD*), pML4 (P_cad_::*proP*), and pML5 (P_cad_::*putP*) confirm OpuC, OpuD, and ProP do not transport proline under these conditions. ^3^H-proline transport data (A) are standardized to JE2 to account for experimental variations and are fit by nonlinear regression using a first-order equation of technical duplicates ran three independent times. Accumulated proline data (B) are represented by the mean ± SD (n = 5–6). Kruskal-Wallis multiple comparisons identified statistically significant differences (* p<0.05). Growth analysis data (C) are represented by the mean ± SD (n = 3).

Despite previous studies identifying ProP, OpuC, and OpuD as potential proline transporters in *S*. *aureus* [17–19,21,24], we were unable to detect proline transport in strains expressing only ProP, OpuD, or OpuC (Fig 2A and 2D). To determine if the lack of proline transport via ProP, OpuC, and OpuD was due to reduced transcription, we introduced a complementation vector encoding each proline transporter with transcription driven by a cadmium-inducible promoter into the penta mutant [28,29]. In agreement with results from strains expressing only a single transporter from its native chromosomal locus (Fig 2), we found that non-cognate expression of PutP and ProT restored accumulation of ^3^H-proline in the penta mutant (Fig 3A and 3B). However, expression of OpuC, OpuD, and ProP in the penta mutant did not result in ^3^H-proline accumulation, showing that, even when expressed from a non-cognate promoter, OpuC, OpuD, and ProP fail to transport proline (Fig 3A). Similar to strains expressing only ProT or PutP (Fig 2A and 2D), the *proT-* and *putP-* complemented strains transported proline, with ProT enabling accumulation of more proline than PutP (Fig 3B). Indeed, growth analysis in CDM documented that the penta mutant expressing either *proT* or *putP* increased growth yield, whereas expression of *opuC*, *opuD*, or *proP* phenocopied growth of the penta mutant with empty vector (Fig 3C). Taken together, these data confirm that *S*. *aureus* encodes two primary proline transporters, PutP and ProT.

### ProT is the primary proline transporter during growth in high salt

**Fig 4:**
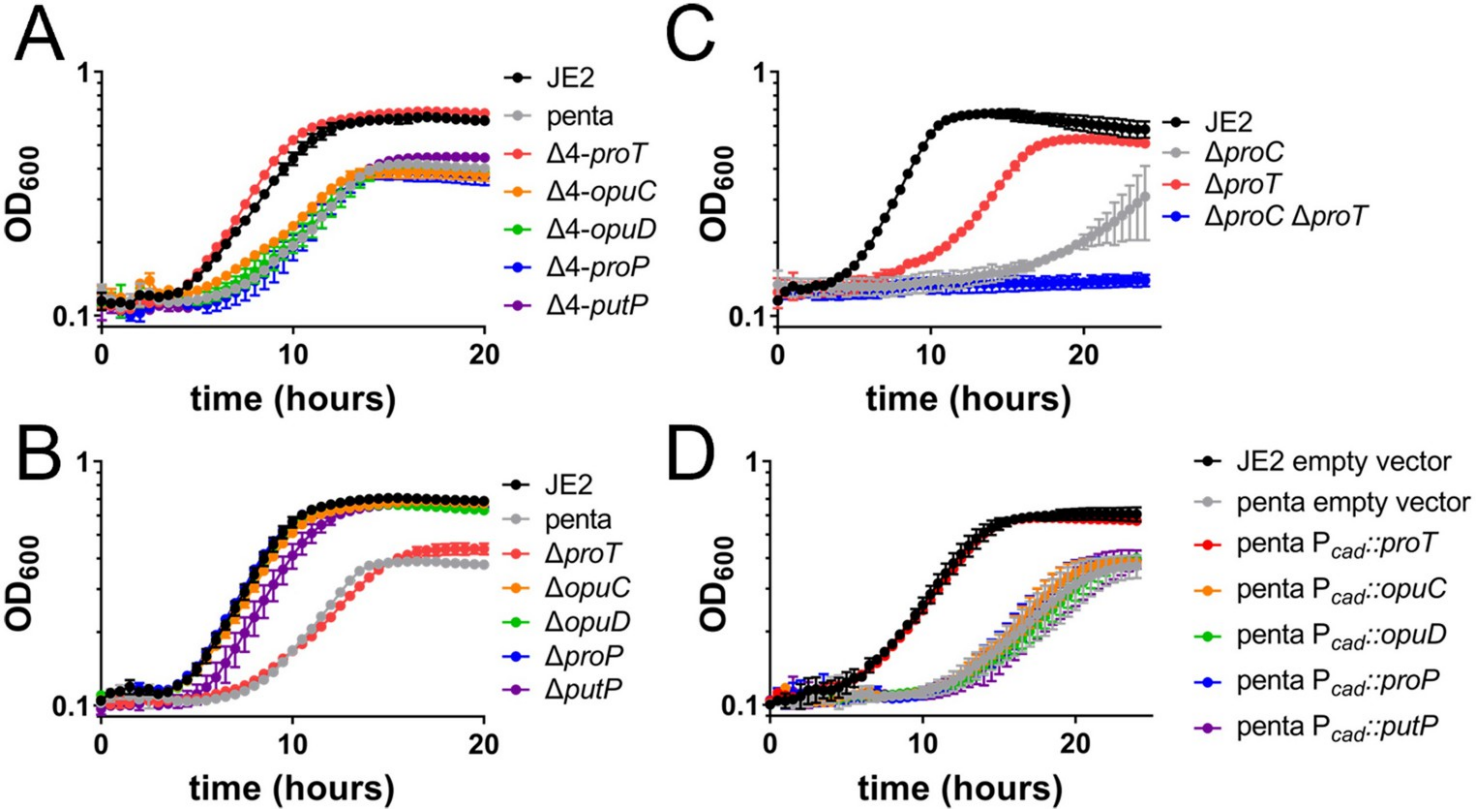
ProT is the primary proline transporter in the presence of high salt. Growth analysis of the following strains in CDM supplemented with 1 M NaCl: A) JE2, penta, Δ4-*proT*, Δ4-*opuC*, Δ4-*opuD*, Δ4-*proP*, and Δ4-*putP* B) JE2, penta, Δ*proT*, Δ*opuC*, Δ*opuD*, Δ*proP*, and Δ*putP* C) JE2, Δ*proC*, Δ*proT*, and Δ*proT* Δ*proC* D) JE2 empty vector, penta empty vector, penta P_cad_::*proT*, penta P_cad_::*opuC*, penta P_cad_::*opuD*, penta P_cad_::*proP*, and penta P_cad_::*putP* reveal ProT is important for maximal growth under these conditions. Data are represented by the mean ± SD (n = 2–3).

Several studies have documented that proline transport is critical for *S*. *aureus* growth during osmotic stress due to its function as an osmolyte [12,13,15,16]. Therefore, growth analyses were performed in CDM containing 1 M NaCl using the various proline transporter mutants (Fig 4). In comparison to JE2, the penta mutant had reduced growth rate and yield in CDM containing 1 M NaCl suggesting that proline transport is beneficial under these growth conditions. (Fig 4A). Moreover, JE2 Δ4-*proT* phenocopied wildtype growth, indicating that ProT is the primary proline transporter that supports growth in high salt (Fig 4A). Surprisingly, growth is still reduced in the Δ4-*putP* strain (Fig 4A), suggesting that PutP- mediated proline transport is not sufficient for maximal growth in high salt. In agreement with these observations, the Δ*proT* single mutant grew similarly to the penta mutant, with diminished growth under 1 M NaCl stress (Fig 4B). All the other single mutants, in which the *proT* allele was still intact, grew similarly to wildtype. Although the growth of the transporter null strain in CDM with 1M NaCl is reduced (Fig 4A and 4B), the fact that it grows suggests that proline biosynthesis is also important under these growth conditions. Indeed, JE2 Δ*proC*, with all five putative proline transporters intact, had diminished growth in CDM with 1 M NaCl (Fig 4C). Moreover, when the *proC* mutation was introduced into JE2 Δ*proT*, growth was completely abrogated, confirming ProT is the primary proline transporter in CDM containing 1 M NaCl (Fig 4C). However, proline synthesis is fundamentally important for growth under these conditions, irrespective of transport. Transcription of *opuD* and *putP* are regulated via the alternative sigma factor σ^B^ [24,26], which regulates the stress regulon including genes that function to allow *S*. *aureus* to grow in medium containing high concentrations of salt [30,31]. However, strains expressing only *opuD* or *putP* did not have any growth advantage over the penta mutant (Fig 4A) suggesting these transporters are also not transporting proline under high salt conditions. Furthermore, when the penta mutant was complemented with each individual proline transporter gene driven by a non-cognate promoter, only *proT* was able to rescue growth in comparison to JE2 (Fig 4D). Overall, proline biosynthesis using arginine as a substrate, in combination with proline transport via ProT, are required for maximal growth under high salt stress.

### Proline transport is important for maximal growth when glucose is present

**Fig 5:**
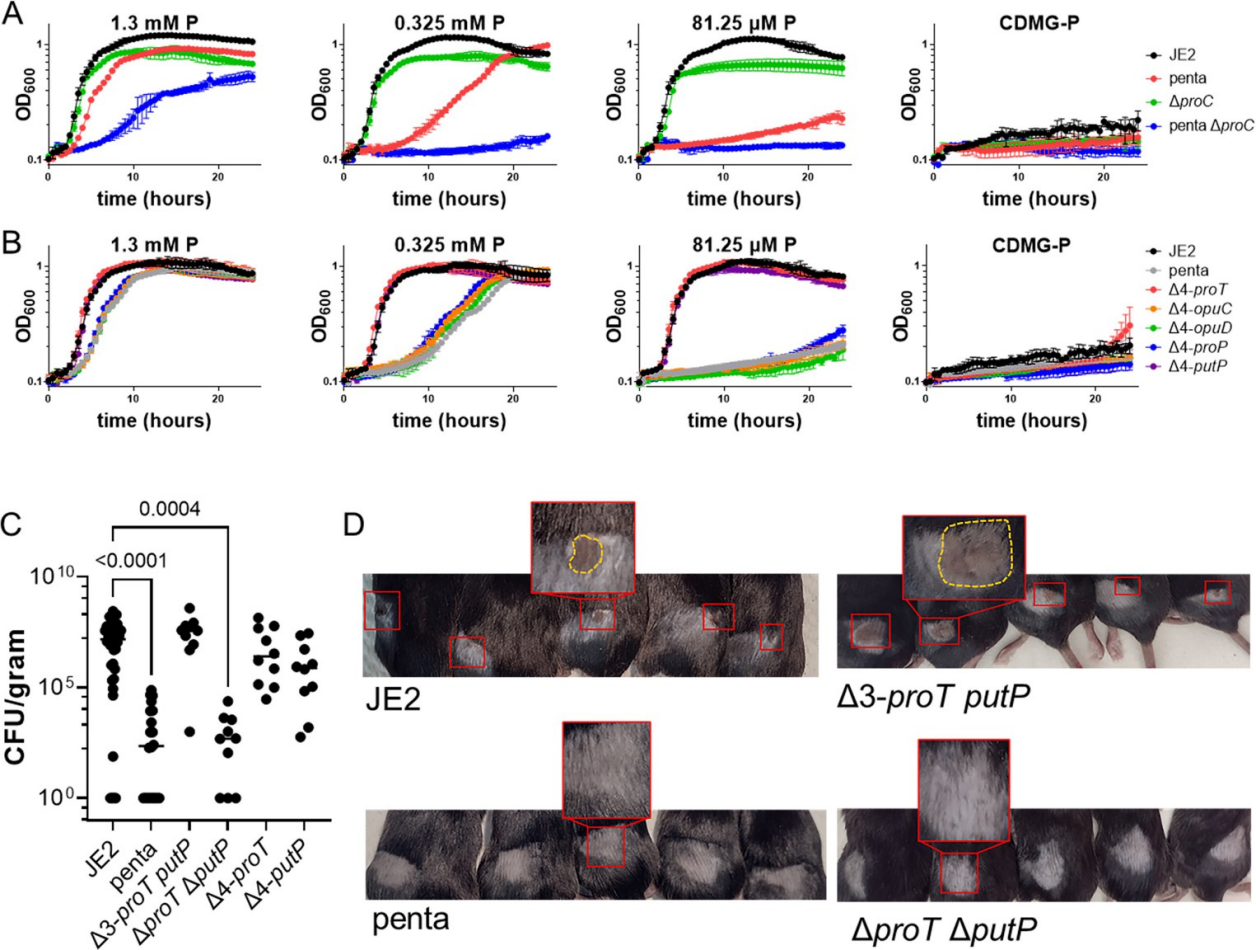
Proline transport supports the establishment of skin and soft tissue infections. A) Growth analysis of JE2, penta, Δ*proC*, and penta Δ*proC* in CDMG (3.5 mM glucose) containing 1.3 mM proline (P), CDMG 0.325 mM P, CDMG 81.25 μM P, or CDMG-P (0 M P). Data are represented by the mean ± SD (n = 3). B) Growth analysis of JE2, penta, Δ4-*proT*, Δ4-*opuC*, Δ4-*opuD*, Δ4-*proP*, and Δ4-*putP* in CDMG (3.5 mM glucose) 1.3 mM P, CDMG 0.325 mM P, CDMG 81.25 μM P, or CDMG-P (0 M P). Data are represented by the mean ± SD (n = 3). C) Bacterial burdens of 7-week-old C57BL/6 mice subcutaneously infected with 1x10^6^ of *S*. *aureus* JE2, penta, Δ3-*proT putP*, Δ*proT* Δ*putP*, Δ4-*proT*, or Δ4-*putP* strain 5 days post-infection. Each datum point represents an individual abscess, with the median represented. Statistical significance was determined by Kruskal-Wallis test, with the p value indicated on the graph. D) Representative images of lesions on 7-week-old C57BL/6 mice subcutaneously infected with 1x10^6^ of *S*. *aureus* JE2, penta, Δ3-*proT putP*, or Δ*proT* Δ*putP* strain 5 days post-infection. Areas outlined in red indicate the area where the mouse was inoculated in the shaved flank. Enlarged image shows a representation of the disease progression observed in the animal, with yellow highlighting the observed lesion.

As previously documented, glucose catabolism and thus CcpA repression functions to repress biosynthesis of proline [5]. Indeed, growth of *S*. *aureus* in CDM containing glucose (CDMG) but lacking proline (CDMG-P) resulted in no growth (Fig 5A). With this observation, we hypothesized that the penta mutant would be unable to grow in CDMG. Surprisingly, the penta mutant grew in CDMG (Fig 5A) although with a slight, reproducible elongation of lag phase. Note, CDMG contains the same amount of proline (1.3 mM) as CDM used in the previously described experiments (Figs 1–4). These data suggest that during growth in CDMG, the penta mutant is either synthesizing or transporting proline using an uncharacterized transport mechanism. Since the presence of glucose represses proline biosynthesis due to repression of *rocF* and *rocD* transcription (S1C Fig) [11], it would be unexpected if this phenotype was partly due to biosynthesis of proline. Nevertheless, growth yield of the penta Δ*proC* mutant was reduced even further in CDMG, suggesting that biosynthesis of proline is partly responsible for growth of the penta mutant in CDMG (Fig 5A). These data also suggest that proline is transported by an unknown transporter. In contrast to wildtype, reducing the proline concentration 4- and 16-fold, further reduced growth of the penta mutant, and minimal growth was noted when 81.25 μM proline was used in CDMG (Fig 5A). The penta Δ*proC* mutant was unable to grow in 0.325 mM and 81.25 μM proline, indicating that the growth of the penta mutant at these proline concentrations was due to biosynthesis, not transport. To determine which proline transporters were required for maximal growth in CDMG, strains expressing single proline transporters were grown in CDMG containing 1.3 mM, 0.325 mM, and 81.25 μM proline. Strains expressing either *proT* or *putP* phenocopied growth of JE2, but strains expressing *opuC*, *opuD*, and *proP* phenocopied growth of the penta mutant (Fig 5B). Use of complementation vectors constitutively expressing each proline transporter confirmed this finding (S4 Fig). Notably, we did observe some growth, albeit greatly delayed, in the penta mutant complemented with *proP* driven by a cadmium inducible promoter, but not single expression strains driven by the native promoter (Δ4-*proP*) (S4 Fig). Taken together, these data reveal that ProT and PutP are important proline transporters for maximal growth when glucose is present. However, an unidentified proline transporter may exist that is utilized when *S*. *aureus* is in the presence of glucose.

### Proline transport is important for establishing a skin and soft tissue abscess

Previous studies have revealed that glycolysis is required to form a skin abscess [2]. When glucose is available, CCR limits the expression of many pathways, including the biosynthesis and catabolism of arginine and proline [4,5]. Therefore, we predicted that JE2 Δ*proC* would not result in a bacterial burden phenotype in a murine skin and soft tissue model, as proline biosynthesis is repressed in the presence of glucose [5,11]. Indeed, no difference in bacterial burden was noted between JE2 Δ*proC* and JE2 (S5 Fig). Similarly, no difference in bacterial burden was observed using JE2 Δ*argG* and JE2 Δ*putA* in comparison to JE2, as arginine biosynthesis via proline is repressed by CCR (S5 Fig) [4]. These data suggest that proline required by *S*. *aureus* for growth must be acquired from the infection milieu, and, therefore, we hypothesized that proline transporters are required for *S*. *aureus* to establish a skin and soft tissue abscess. Indeed, mice infected subcutaneously with the penta mutant had a significant reduction in bacterial burden (~5 log_10_) as compared to JE2 (Fig 5C). Notably, as compared to mice infected with JE2, mice infected with the penta mutant lacked visible necrosis or abscess (Fig 5D). To determine which of the transporters are responsible for proline transport in this model, the Δ3-*proT putP* and Δ*proT* Δ*putP* strains were first used to determine the contribution, if any, of OpuC, OpuD, and ProP. Unsurprisingly, bacterial burden in the Δ3-*proT putP* strain was similar to JE2, suggesting the expression of these two proline transporters is sufficient to support growth and establish a skin abscess (Fig 5C and 5D). Conversely, the Δ*proT* Δ*putP* strain had severely reduced bacterial burden in the skin abscess model, similar to the penta mutant, demonstrating that both strains are unable to establish an infection (Fig 5C and 5D). Single expression strains were then tested to determine if either ProT or PutP were essential for establishment of a skin abscess. Infection with both the Δ4-*proT* and Δ4-*putP* strains resulted in similar pathology and bacterial burden as wildtype documenting that both ProT and PutP mediate proline transport sufficient to support infection in a murine skin and soft tissue model (Fig 5B). Similarly, a significantly reduced bacterial burden was observed in the kidney and liver when the penta and Δ*proT* Δ*putP* mutants were compared to JE2 in a murine bacteremia model (S6 Fig). Additionally, in the bacteremia model, the Δ4-*proT* and Δ4-*putP* strains had bacterial burdens similar to wildtype. Taken together, these data confirm that ProT and PutP function as the primary proline transporters in *S*. *aureus* and can complement one another during growth in murine models of infection.

## Discussion

These studies reveal that *S*. *aureus* encodes a previously uncharacterized proline transporter, ProT, that is functional under all conditions tested. Additionally, a second proline transporter, PutP, is also important for proline transport except under conditions of high salinity. Due to its multiple functions in the cell, it is not surprising that *S*. *aureus* encodes redundant systems to acquire proline [14]. In a skin and soft tissue infection, we observed the effects of this redundancy, with both ProT and PutP able to support growth. When proline transport is abolished, abscess formation is greatly diminished (Fig 5C and 5D), indicating that proline acquisition is critical in an *in vivo* environment.

There is growing evidence that *S*. *aureus* encounters spatiotemporal nutrient requirements during infection. For example, during the establishment of an infection, fermentation, mediated by the glucose-induced *ldh1* allele, facilitates the growth of *S*. *aureus* in the presence of the innate immune radical nitric oxide (NO^.^) [2]. Further, mice infected with *S*. *aureus* containing mutations that inactivate glycolysis result in a significant attenuation of infection, with little organ colonization and reduced bacterial burden, abscess, and lesion formation in the skin abscess. Similarly, in mice infected with the proline transporter mutants, we observed dramatic attenuation in skin abscess formation, bacterial burden, and organ colonization (Figs 5C and 5D, and S6), suggesting that proline transport is imperative for bacterial survival during the early stages of infection. We hypothesize this is likely due to the CcpA mediated repression of proline biosynthesis pathways (S1C Fig), and therefore requiring exogenous proline to be transported as a proteinogenic substrate. These findings correlate with our observations of growth in CDMG (Fig 5B). In CDMG containing high proline concentrations (1.3 mM), proline transport was observed via transporters not characterized in this study, suggesting there may be low affinity and/or promiscuous permeases that allow sufficient proline transport to support growth when proline concentrations in the medium is very high. Growth at lower proline concentrations (≤325 μM) revealed that any growth observed was due to proline biosynthesis, not proline transport. This finding was unexpected as proline biosynthesis is regulated via CCR and suggests that lack of proline transport may provide regulatory feedback to proline biosynthetic pathways. Furthermore, when glucose was present, either ProT or PutP were sufficient for accumulation of enough proline to support maximal growth. Notably, normal human plasma levels of proline range between 77.8–272.7 μM [32], suggesting that the conditions encountered during the initial stages of an infection likely require proline transport via ProT and/or PutP for maximal growth. Again, this correlates with the results of our *in vivo* studies which revealed that proline transport is required for establishing an infection.

In the initial studies characterizing proline transport in *S*. *aureus*, two proline transport systems (high- and low-affinity) were identified [17,19,33]. These initial observations correlated with the information available on those systems studied in both *E*. *coli* and *Salmonella enterica* serotype Typhimurium, which both encode a high-affinity transporter, PutP, and two low-affinity transporters, ProP and ProU [34–36]. Later studies identified a PutP ortholog in *S*. *aureus* [22] that when inactivated, reduced *S*. *aureus* proline accumulation by approximately 33% and resulted in reduced pathogenesis in a burn wound, endocarditis, bacteremia, and skin and soft tissue infection [23,25,26]. Although a ProP ortholog has been identified in *S*. *aureus*, no studies have been performed to determine its function with regards to proline transport [21]. Our studies have shown that, at least under the conditions studied, ProP does not function as a primary proline transporter. OpuD is another low-affinity proline transporter described in *S*. *aureus* that is orthologous to a low affinity transporter previously described in *B*. *subtilis* [24]. ^3^H-proline transport studies of an *opuD* mutant performed in low (2.5 μM) and high (400 μM) proline concentrations revealed reduced transport at high proline concentrations [24]. Furthermore, similar to our studies, an *opuD* mutant did not affect *S*. *aureus* survival in a skin and soft tissue infection. More recently, it has been reported that OpuD is a glycine betaine transporter [37], suggesting that glycine betaine transport may be the primary function of OpuD, with proline transport only occurring at very high proline concentrations. This correlates with the early studies of osmolyte transporters in *S*. *aureus* that also revealed high- and low- affinity glycine betaine transport systems, with the low-affinity glycine betaine transporter also capable of transporting proline [18]. It was surmised that the previously reported low-affinity proline transporter [19,22] and glycine betaine transporter were likely the same transporter. Currently there is no direct evidence suggesting that OpuC is a proline transporter, although it is a characterized carnitine transporter [20], with speculation that, as an osmolyte transporter, it may also be a low-affinity proline transporter similar to OpuD [21]. Although proline transport was not dependent upon ProP, OpuD, or OpuC under the conditions tested throughout these experiments, it is possible that they may transport proline at very high concentrations. Nevertheless, we did not observe a biological function of these low-affinity transporters in our *in vivo* models of infection.

The initial studies of proline transport also found that proline transport was Na^+^ dependent [17,19]. This correlates with the observation that PutP and ProT, two members of the Na^+^/solute symporter family (TC 2A.21, SLC5) [38–40], are the primary proline transporters in *S*. *aureus*. Although some Na^+^ is required for proline transport, growth analysis under high salinity indicates that the newly identified transporter, ProT, but not PutP, is key for optimal growth of *S*. *aureus* (Fig 4). This suggests that PutP is not important for proline accumulation under high-salt conditions. Furthermore, despite previous studies indicating that *putP* transcription increased under high osmotic stress, but low proline concentrations [26], PutP did not appear to have a function in proline transport under high saline conditions even when expressed by a non-cognate promoter (Fig 4D). This suggests that transcription is not limiting the transport of proline via PutP under these conditions, but rather the conditions are likely hindering the function of the transporter.

*S*. *aureus* has evolved to utilize a metabolic repertoire that facilitates colonization or infection of virtually any human niche. A model is proposed whereby amino acid transporters are essential for establishing an infection if amino acid biosynthetic pathways are repressed by CcpA. Indeed, the presence of glucose, and thus CcpA, represses both proline and arginine biosynthesis and catabolism [2,4,5]. However, once an abscess is established, glucose and arginine are depleted due to rapid consumption by host immune cells under the oxygen-limiting conditions [2,4,6–10,41,42], requiring the bacteria to acquire secondary carbon sources, such as free amino acids and peptides, for proliferation [6,11]. Thus, even though glucose is depleted and CcpA repression should be alleviated, the lack of free arginine, which is required for proline biosynthesis, may suggest that proline is acquired via PutP/ProT or through peptide acquisition via Opp3 [6].

## Materials and methods

### Ethics statement

Animal experiments were performed in ABSL2 facilities in accordance with protocols (21-002-11-FC and 21-003-11-FC) approved by the Institutional Animal Care and Use Committee (IACUC). All animals at the University of Nebraska Medical Center are maintained in compliance with the Animal Welfare Act and the Department of Health and Human Service “Guide for the Care and Use of Laboratory Animals.”

### Strains, plasmids, and growth conditions

All strains and plasmids used in these studies are listed in S1 Table. Defined *bursa aurealis* transposon mutants were obtained from the Nebraska Transposon Mutant Library and backcrossed to JE2 using Φ11 [43]. Different transposon mutant strains had the antibiotic markers altered to accommodate transduction of multiple mutations into one strain as previously described [44]. The Δ*putP* allelic replacement mutant was constructed by cloning 1 kb fragments upstream and downstream of the open reading frame (ORF) into pJB38. The ORF was replaced with SacI recognition sequence. Similarly, the Δ*proT* and Δ*opuD* allelic replacement mutants were also generated by cloning 1 kb fragments upstream and downstream of the ORF with trimethoprim and erythromycin resistance cassettes to replace the ORF, respectively (see S2 Table for details). Overnight cultures of bacteria were grown at 37° shaking at 250 rpm in 5 ml Tryptic Soy Broth (TSB) in the presence of chloramphenicol (10 μg ml^-1^) as needed. Growth curves of biological replicates were performed in CDM with no glucose [45] or CDM with 3.5 mM glucose (CDMG) in 96-well plates in the TECAN InfinitePro (TECAN), at 37° shaking at 250 rpm. The mean in the data presented depict one representative experiment, performed at least three independent times. Unless noted in the legends, the standard amount of proline added to CDM or CDMG is 1.3 mM [11]. The P_*cad*_::*proT*, P_*cad*_::*opuC*, P_*cad*_::*opuD*, P_*cad*_::*proP*, and P_*cad*_::*putP* plasmids, pML1, pML2, pML3, pML4, and pML5, respectfully, were created by amplifying the entire ORF of each gene from JE2 chromosomal DNA with the respective primers listed in S2 Table, and cloned into the PstI and SalI sites of pBK123 [28,29]. Growth CDM or CDMG with the strains with plasmids included 1 μg ml^-1^ chloramphenicol for retention of the plasmid and 100 nM cadmium chloride to induce expression.

### Amino acid consumption assay

JE2, Δ*opuC* Δ*opuD* Δ*proP* Δ*putP*, and DHP-resistant strains (DHP-1, DHP-2, DHP-3, and DHP4) were grown in 25 ml TSB in a 250 ml flask, with a starting OD_600_ = 0.05. After the cells reached stationary phase, the cells were pelleted, and supernatant was collected and filtered through Amicon Ultra centrifugal filters (Millipore) (3,000 molecular weight cutoff) according to the manufacturer’s protocol. Amino acid analysis was performed by the Protein Structure Core Facility, University of Nebraska Medical Center (UNMC), using a Hitachi L-8800 amino acid analyzer.

### Toxic analog screen

From a 5 ml overnight culture of JE2 Δ*opuC* Δ*opuD* Δ*proP* Δ*putP* grown in TSB, 1 ml was centrifuged and washed twice with Dulbecco’s Phosphate Buffered Saline (DPBS) and diluted to an OD_600_ = 1 in DPBS. This was used as the starting inoculum for the lawn of bacteria swabbed onto a modified CDM lacking proline (CDM-P) agar (1%) plate. The modified CDM-P is standard CDM with 5x excess arginine, serine, threonine, glycine, and alanine added to support robust growth. A 6mM sterile disk (Becton Dickinson) was embedded with 15 μl of 100 mg ml^-1^ 3,4-dehydroproline (Sigma) and placed in the center of the plate. The plates were placed at 37° for 16+ hours and photographed. Resistant cells selected in the zone of inhibition were re-isolated on TSA and screened as described above for resistance to DHP.

### Whole genome sequencing

Genomic DNA of 4 JE2 isolates was prepared for sequencing using the Nextera XT DNA Library Prep kit (Illumina) and associated protocol. Libraries were validated by running 5ul of PCR cleanup on a 1% agarose gel, then bead-normalized and pooled in equal volumes. Pooled normalized libraries (2 nM starting concentration assumed) and PhiX were diluted and denatured according to the MiSeq System Users Guide, with a final concentration of 80 pM. The final pool was heated at 96°C for 3 min to ensure denaturation before sequencing on a MiSeq using read length 2x300bp, and onboard fastq file generation and sample demultiplexing, generating 0.6–1.4 million paired reads per sample. Reads were processed using CLC Genomics Workbench (v. 20.0.4) and the Microbial Genomics Module (v. 20.1.1) “Type a Known Species” workflow. Reads were also mapped to the JE2 chromosome to identify single and multi-nucleotide variants relative to the laboratory strain. Note, the amino acid in which the change occurred is identified in parentheses in Table 1. The amino acid location of the frame shift identifies the number of amino acids intact prior to the nucleotide deletion that resulted in a frameshift mutation.

### Radiolabeled-proline transport assay

Overnight cultures of single colonies inoculated into 5 ml TSB were washed with CDM and used to start 50 ml CDM cultures in 500 ml flasks (250 rpm) at an OD_600_ = 0.1. Cells were grown until they reached an OD_600_ = 0.5–1.0. The entire culture (50 ml) was centrifuged at 4,000 rpm in the Sorvall Legend X1R Centrifuge (Thermo Scientific) for 10 minutes. The supernatant was removed, and cells were resuspended in 10 ml CDM-P. The OD_600_ was determined, and the cells were centrifuged again at 4,000 rpm in the Sorvall Legend X1R Centrifuge for 10 minutes. The cells were resuspended in CDM-P to an OD_600_ = 10. Once the cell suspension was prepared, 100 μl were aliquoted into microcentrifuge tubes and placed on ice. For the assay, 900 μl of pre-warmed (37°) CDM-P was added to 100 μl OD_600_ = 10 cells for a final OD_600_ = 1 (10^8^ CFU). For the blank, 100 μl of the sample was collected on 25 mM diameter 0.45 μM nitrocellulose membrane (Whatman) and washed with 10 ml DPBS with 10 mM proline. The remaining 900 μl of sample was added to pre-warmed 1 mM proline (0.1 mCi (L-[2,3,4,5-^3^H]-Proline)/1mM proline) (Perkin Elmer) to give a final concentration of 0.1 mM proline for the assay, and incubated at 37° C. Samples (100 μl) were collected at timed intervals (e.g., 30 seconds, 1 minute, 2 minutes, 4 minutes, 6 minutes, 8 minutes, and 10 minutes) after addition of the bacteria to the radiolabeled proline. All samples were immediately applied to a 25 mM diameter 0.45 μM nitrocellulose membrane with vacuum and washed with 10 ml DPBS containing 10 mM proline. Bacteria on the washed filters were placed into scintillation tubes and allowed to dry at least 16 hours. Filter Count scintillation fluid (Perkin Elmer) was added to the samples. The filters were allowed to dissolve over 16 hours in the scintillation fluid, and then counted using a Packard Tri-Carb 2910TR scintillation counter to determine the CPM per sample. The transport assay curves were fit by non-linear regression using a first-order equation ((Pro = A*(1-exp(-B*Time))). Data was normalized to JE2 to account for experimental variations.

### Animal studies

7-week-old C57B/6 mice (Charles River Laboratories, Wilmington, MA) were inoculated subcutaneously with 1 x 10^6^ CFU *S*. *aureus*. Prior to inoculation, one flank of each mouse was shaved and swabbed with 70% alcohol. Mice were sacrificed on day 5 post-inoculation, abscess and surrounding tissue was removed and placed in DPBS to be homogenized and analyzed for CFU analysis. For the bacteremia studies, 5 x 10^6^ CFU *S*. *aureus* were injected retro-orbitally. Mice were sacrificed 5 days post-infection. These studies were conducted in accordance with the recommendations in the *Guide for the Care and Use of Laboratory Animals* of the National Institutes of Health. The animal protocol was approved by the Institutional Animal Care and Use Committee of the University of Nebraska Medical Center (Protocols 21-002-11-FC and 21-003-11-FC).

### Data deposition

The numerical data used in all figures can be found in S1 Data.

## Supporting information

S1 Fig Selection of Isolates Resistant to the Toxic Proline Analog 3,4 dehydro-DL-proline (DHP). A) A lawn of JE2 Δ*opuC* Δ*opuD* Δ*proP* Δ*putP* was struck on modified CDM-P agar (see methods and materials). A sterile disk imbedded with 2 μg of DHP was placed in the center and incubated for 18 hours. Colonies in the zone of inhibition (left image) were isolated and subcultured on Tryptic Soy Agar (TSA). Isolates obtained from the zone of inhibition were fully resistant to DHP (right image; DHP-4) suggesting they were unable to transport DHP. Growth was supported on CDM-P (CDM lacking proline) agar through the biosynthesis of proline via ProC. B) JE2, JE2 Δ*opuC* Δ*opuD* Δ*proP* Δ*putP*, and DHP 1–4 were grown to stationary phase in 25 ml TSB in a 250 ml flask and amino acid consumption assays were performed on the spent medium. Note a lack of proline consumption in DHP 1–4 in comparison to WT JE2 and JE2 Δ*opuC* Δ*opuD* Δ*proP* Δ*putP*. We hypothesize that the accumulation of proline the DHP resistant strains is due to proteolysis of peptides in the TSB. C) Proline and arginine biosynthetic pathways in *S*. *aureus*. Arginine serves as a substrate for proline biosynthesis via RocF, RocD, and ProC. Proline serves as a substrate for arginine biosynthesis via PutA, RocD, ArcB1, ArgG and ArgH. The transcription of *putA*, *rocD*, *arcB1*, *argG*, *argH*, *and rocF* are repressed by CcpA. This is adapted from Reslane et al. and Halsey et al.
(TIF)

S2 Fig Redundancy in proline transport. Growth curve analysis of JE2, penta, Δ*B7H15_03660* (Δ*03660*), Δ*opuC*, Δ*opuD*, Δ*proP*, and Δ*putP* in CDM reveal *S*. *aureus* encodes multiple proline transporters. Data are represented by the mean ± SD (n = 3).
(TIF)

S3 Fig ProT is highly conserved among staphylococcal species. ProT orthologues from representative staphylococcal species were aligned using Jalview 2.11.2.4. The referenced alleles include: ARG45194.1 (*S*. *aureus*), WP_207517961.1 (*S*. *simiae*), WP_064212334.1 (*S*. *capitis*), WP_251481646.1 (*S*. *xylosus*), WP_087436598.1 (*S*. *hominis*), WP_107614115.1 (*S*. *haemolyticus*), WP_150873555.1 *(S*. *saprophyticus*), and WP_107511203.1 (*S*. *epidermidis*).
(TIF)

S4 Fig Proline transport is essential for growth in CDMG. Growth analysis of JE2 and penta pBK123 (empty vector), pML1 (P_cad_::*proT*), pML2 (P_cad_::*opuC*), pML3 (P_cad_::*opuD*), pML4 (P_cad_::*proP*), and pML5 (P_cad_::*putP*) in CDMG (3.5 mM glucose) 1.3 mM Proline (P), CDMG 0.325 mM P, CDMG 81.25 μM P, or CDMG-P 0 M P confirm transport via ProT and PutP. Data are represented by the mean ± SD (n = 3).
(TIF)

S5 Fig Arginine and proline biosynthesis are not important for pathogenesis of a 5-day skin abscess. Bacterial burdens of 7-week-old C57BL/6 mice subcutaneously infected with 1x10^6^ of *S*. *aureus* JE2, Δ*proC*, Δ*argH*, or Δ*putA* strain 5 days after infection revealed no significant difference in bacterial burden. Data are represented by the median with statistical significance determined by Kruskal-Wallis test.
(TIF)

S6 Fig Proline transport is required for organ colonization in a bacteremia model. Bacterial burdens of 7-week-old C57BL/6 mice retro-orbitally inoculated with 5x10^6^ of *S*. *aureus* JE2, penta mutant, Δ*proT* Δ*putP*, Δ4-*proT*, and Δ4-*putP* 5 days post-infection in the kidneys and liver were determined. Data are represented by the median with statistical significance determined by Mann-Whitney test. p values are defined on the graph.
(TIF)

S1 Table Strains and plasmids used in these studies. (DOCX)

S2 Table Primers used in these studies. (DOCX)

S1 Data Numerical data for figures. (XLSX)
