# Supplementary figures and images for: Proline transporters ProT and PutP are required for Staphylococcus aureus infection
Source: PLoS Pathog. 2023 Jan 18;19(1):e1011098. doi: 10.1371/journal.ppat.1011098 (PMC9886301; doi:10.1371/journal.ppat.1011098)

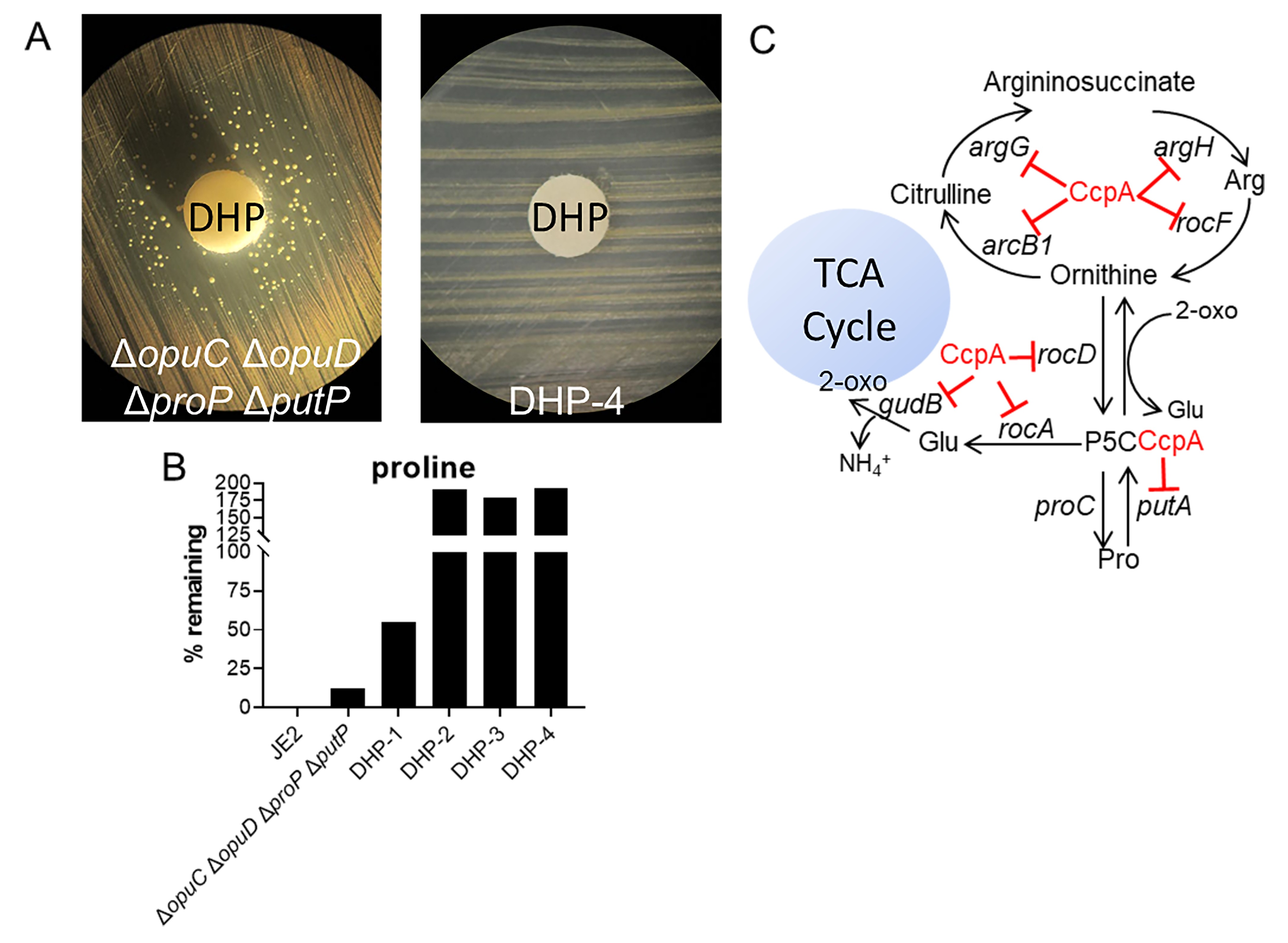

Supplement: S1 Fig — A) A lawn of JE2 ΔopuC ΔopuD ΔproP ΔputP was struck on modified CDM-P agar (see methods and materials). A sterile disk imbedded with 2 μg of DHP was placed in the center and incubated for 18 hours. Colonies in the zone of inhibition (left image) were isolated and subcultured on Tryptic Soy Agar (TSA). Isolates obtained from the zone of inhibition were fully resistant to DHP (right image; DHP-4) suggesting they were unable to transport DHP. Growth was supported on CDM-P (CDM lacking proline) agar through the biosynthesis of proline via ProC. B) JE2, JE2 ΔopuC ΔopuD ΔproP ΔputP, and DHP 1–4 were grown to stationary phase in 25 ml TSB in a 250 ml flask and amino acid consumption assays were performed on the spent medium. Note a lack of proline consumption in DHP 1–4 in comparison to WT JE2 and JE2 ΔopuC ΔopuD ΔproP ΔputP. We hypothesize that the accumulation of proline the DHP resistant strains is due to proteolysis of peptides in the TSB. C) Proline and arginine biosynthetic pathways in S. aureus. Arginine serves as a substrate for proline biosynthesis via RocF, RocD, and ProC. Proline serves as a substrate for arginine biosynthesis via PutA, RocD, ArcB1, ArgG and ArgH. The transcription of putA, rocD, arcB1, argG, argH, and rocF are repressed by CcpA. This is adapted from Reslane et al. and Halsey et al. (TIF) [file ppat.1011098.s001.tif]

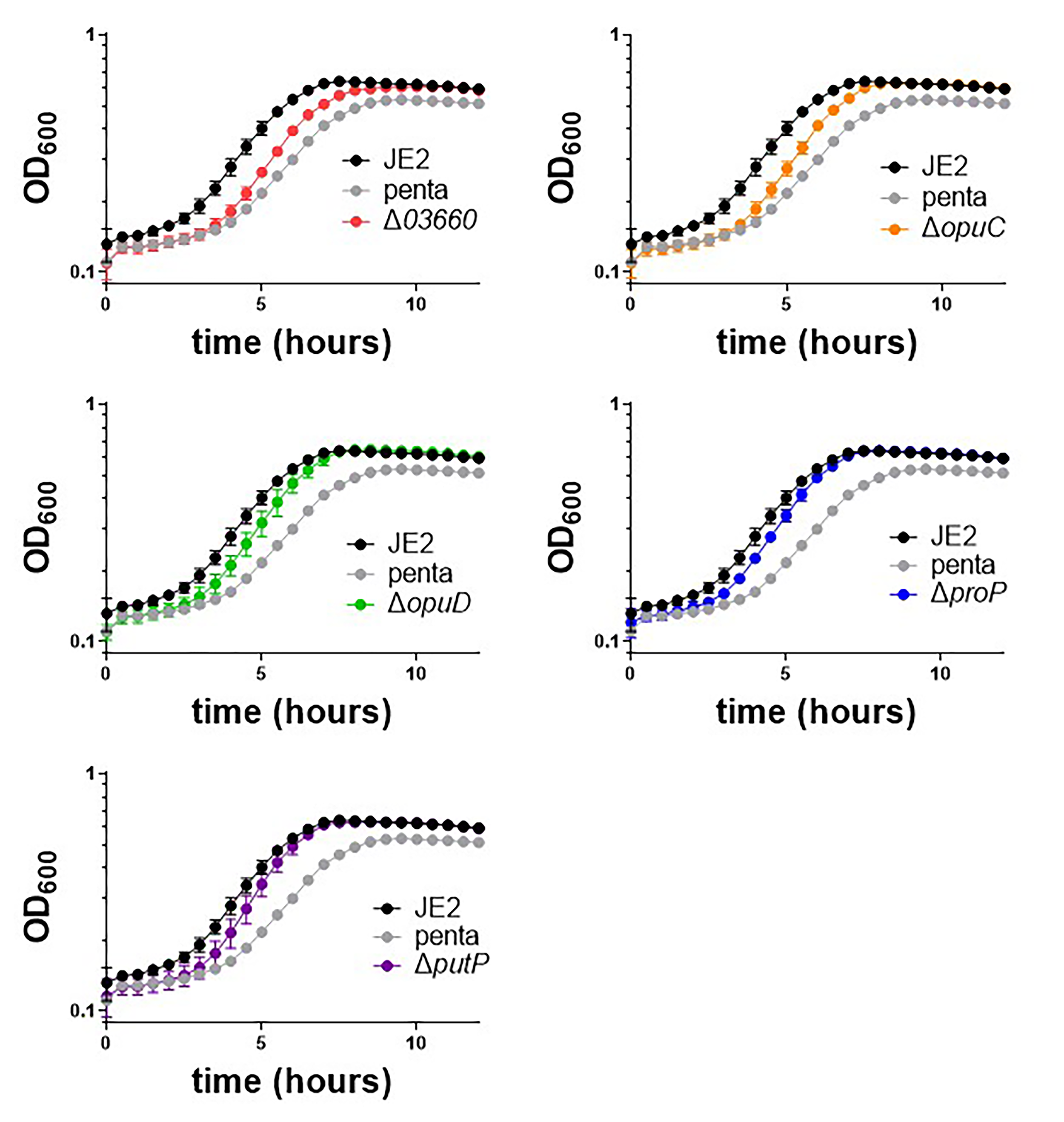

Supplement: S2 Fig — Growth curve analysis of JE2, penta, ΔB7H15_03660 (Δ03660), ΔopuC, ΔopuD, ΔproP, and ΔputP in CDM reveal S. aureus encodes multiple proline transporters. Data are represented by the mean ± SD (n = 3). (TIF) [file ppat.1011098.s002.tif]

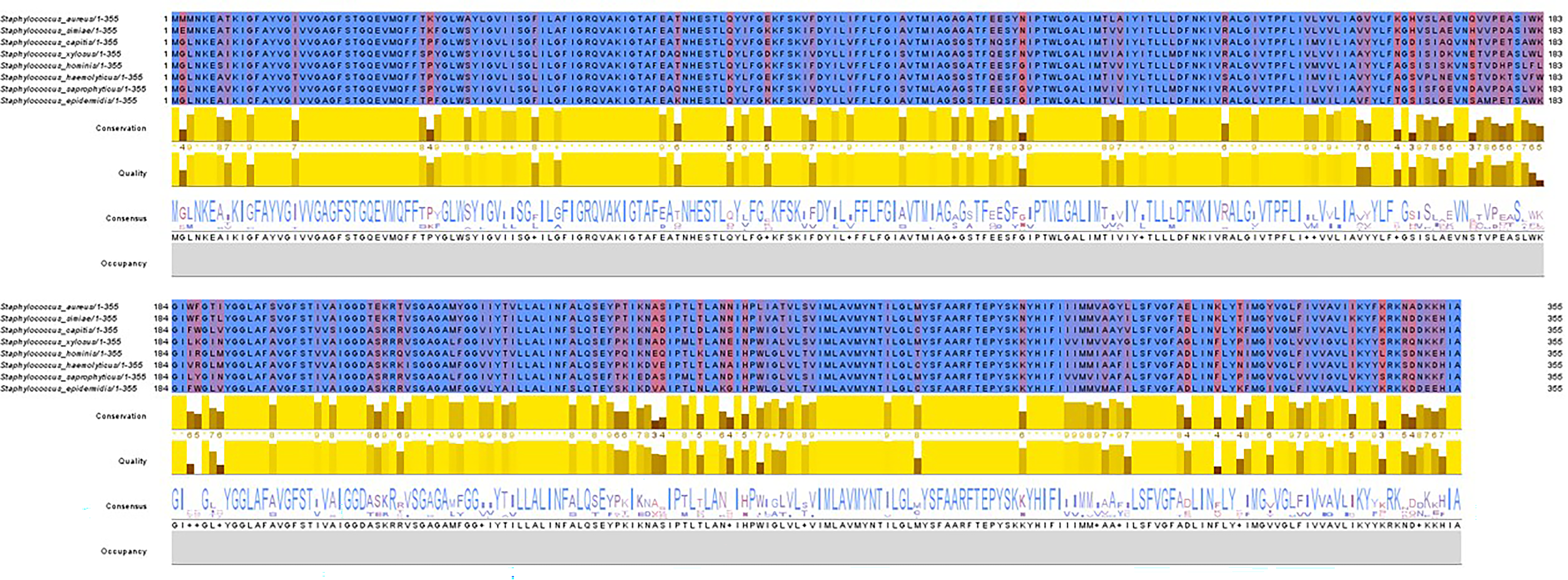

Supplement: S3 Fig — ProT orthologues from representative staphylococcal species were aligned using Jalview 2.11.2.4. The referenced alleles include: ARG45194.1 (S. aureus), WP_207517961.1 (S. simiae), WP_064212334.1 (S. capitis), WP_251481646.1 (S. xylosus), WP_087436598.1 (S. hominis), WP_107614115.1 (S. haemolyticus), WP_150873555.1 (S. saprophyticus), and WP_107511203.1 (S. epidermidis). (TIF) [file ppat.1011098.s003.tif]

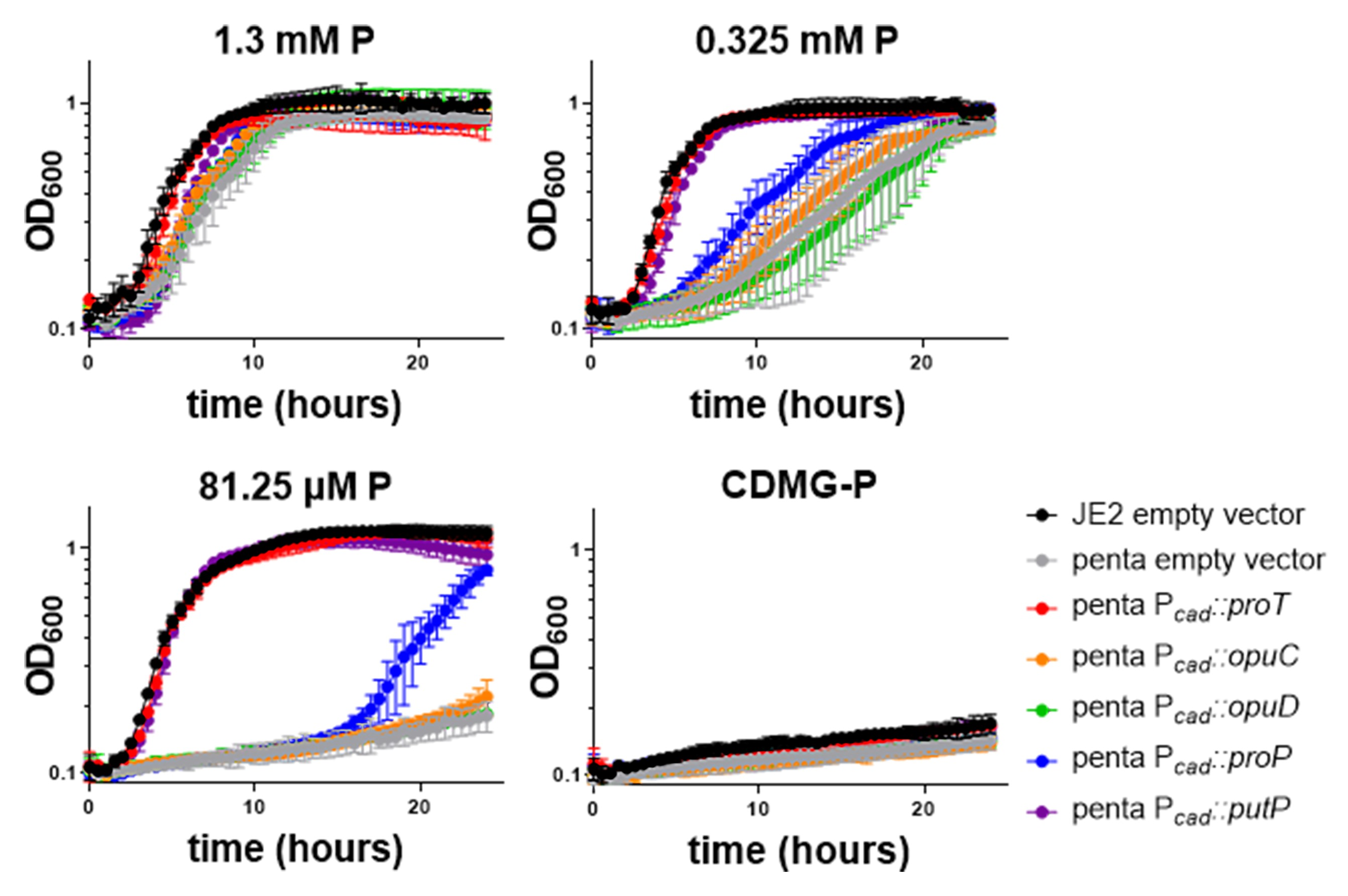

Supplement: S4 Fig — Growth analysis of JE2 and penta pBK123 (empty vector), pML1 (Pcad::proT), pML2 (Pcad::opuC), pML3 (Pcad::opuD), pML4 (Pcad::proP), and pML5 (Pcad::putP) in CDMG (3.5 mM glucose) 1.3 mM Proline (P), CDMG 0.325 mM P, CDMG 81.25 μM P, or CDMG-P 0 M P confirm transport via ProT and PutP. Data are represented by the mean ± SD (n = 3). (TIF) [file ppat.1011098.s004.tif]

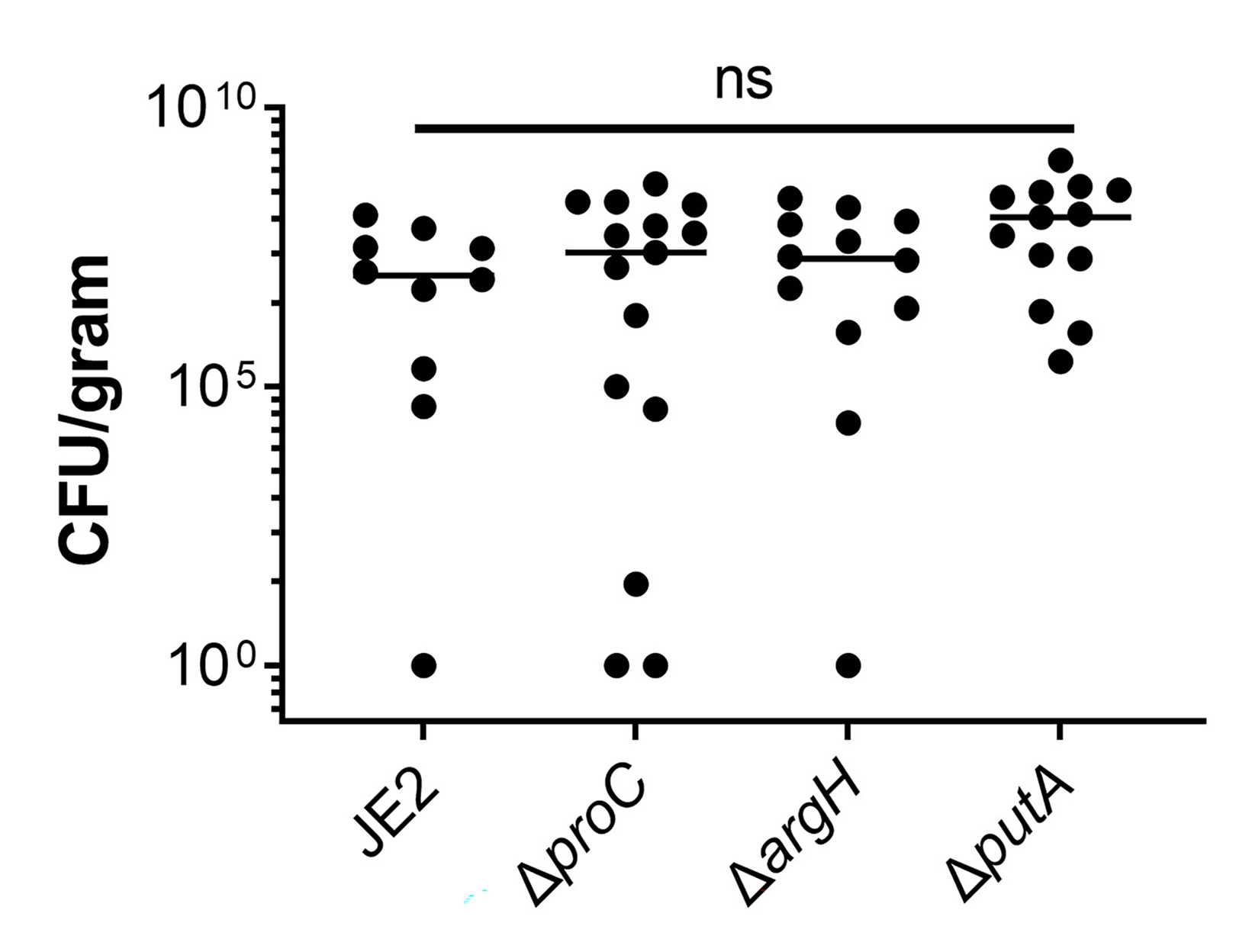

Supplement: S5 Fig — Bacterial burdens of 7-week-old C57BL/6 mice subcutaneously infected with 1x10^6^ of S. aureus JE2, ΔproC, ΔargH, or ΔputA strain 5 days after infection revealed no significant difference in bacterial burden. Data are represented by the median with statistical significance determined by Kruskal-Wallis test. (TIF) [file ppat.1011098.s005.tif]

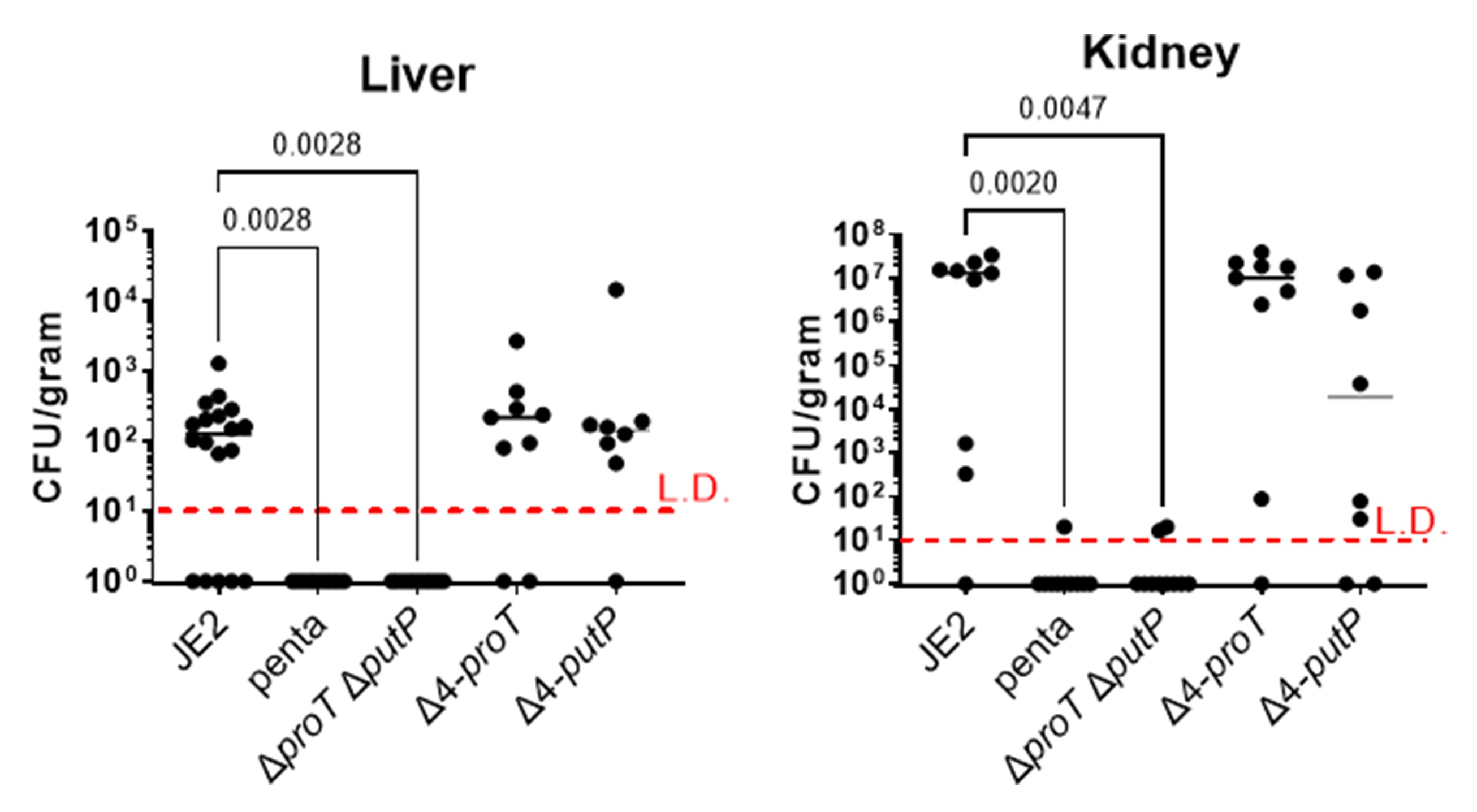

Supplement: S6 Fig — Bacterial burdens of 7-week-old C57BL/6 mice retro-orbitally inoculated with 5x10^6^ of S. aureus JE2, penta mutant, ΔproT ΔputP, Δ4-proT, and Δ4-putP 5 days post-infection in the kidneys and liver were determined. Data are represented by the median with statistical significance determined by Mann-Whitney test. p values are defined on the graph. (TIF) [file ppat.1011098.s006.tif]
