# Supplementary material for: Proline transporters ProT and PutP are required for Staphylococcus aureus infection
Source: PLoS Pathog. 2023 Jan 18;19(1):e1011098. doi: 10.1371/journal.ppat.1011098 (PMC9886301; doi:10.1371/journal.ppat.1011098)
Supplement: S1 Table — (DOCX) [file ppat.1011098.s007.docx]

**S1 Table. Strains and plasmids used in these studies.**

| **Bacterial Strain or plasmid** | **Relevant phenotype** | **Source** |
| --- | --- | --- |
| *S. aureus* JE2 | Wildtype USA300 isolate | (1) |
| JE2 *proC::kan* (Δ*proC*) | *proC::ΦΝΕ* with erythromycin resistance replaced with kanamycin resistance | This study |
| JE2 Δ*putP* (Δ*putP*) | Allelic replacement of *putP* ORF | This study |
| JE2 *proT::dhfR* (Δ*proT*) | Allelic replacement of *proT* ORF with trimethoprim resistance cassette | This study |
| JE2 *proP::specT* (Δ*proP*) | *proP::ΦΝΕ* with erythromycin resistance replaced with spectinomycin resistance | This study |
| JE2 *opuCa::tet* (Δ*opuC*) | *opuCa::ΦΝΕ* with erythromycin resistance replaced with tetracycline resistance | This study |
| JE2 *opuD::erm* (Δ*opuD*) | Allelic replacement of *opuD* ORF with *ermD* | This study |
| JE2 *proC::kan proT::dhfR* (Δ*proC* Δ*proT*) | *proT::dhfR* transduced into *proC::kan* via Φ11 | This study |
| JE2 Δ*putP proT::dhfR proP::specT opuCa::tet opuD::erm* (penta) | *proT::dhfR* transduced into quad +*proT* | This study |
| JE2 *proC::kan* Δ*putP proT::dhfR proP::specT opuCa::tet opuD::erm* (penta Δ*proC*) | *proC::kan* transduced into penta via Φ11 | This study |
| JE2 Δ*putP proT::dhfR proP::specT opuCa::tet* (Δ4-*opuD*) | *proP::specT* and *opuCa::tet* transduced into Δ*proT* Δ*putP* via Φ11 | This study |
| JE2 Δ*putP proT::dhfR proP::specT opuD::erm* (Δ4-*opuC*) | *opuD::erm* and *proP::specT* transduced into Δ*proT* Δ*putP* via Φ11 | This study |
| JE2 Δ*putP proT::dhfR opuCa::tet opuD::erm* (Δ4-*proP*) | *opuCa::tet* and *opuD::erm* transduced into Δ*proT* Δ*putP* via Φ11 | This study |
| JE2 Δ*putP proP::specT opuCa::tet opuD::erm* (Δ4-*proT*) | *opuCa::tet* transduced into Δ*putP* Δ*proP* Δ*opuD* via Φ11 | This study |
| JE2 *proT::dhfR proP::specT opuCa::tet opuD::erm* (Δ4-*putP*) | *proT::dhfR* transduced into Δ*proP* Δ*opuC* Δ*opuD* via Φ11 | This study |
| JE2 *proT::dhfR* Δ*putP* (Δ*proT* Δ*putP*) | *proT::dhfR* transduced into Δ*putP* via Φ11 | This study |
| JE2 *proP::specT opuCa::tet opuD::erm* (Δ3-*proT putP*) | *opuCa::tet* transduced into Δ*proP* Δ*opuD* via Φ11 | This study |
| pBK123 (empty vector) | Chloramphenicol-resistant derivative of shuttle plasmid pCN51 | (2, 3) |
| pML1 (P*_cad_::proT*) | pBK123 with the *proT* ORF cloned behind the cadmium-inducible promoter at the *Sal*I/*Xma*I sites | This study |
| pML2 (P*_cad_::opuC*) | pBK123 with the *opuCabcd* operon ORF cloned behind the cadmium-inducible promoter at the *Sal*I/*Xma*I sites | This study |
| pML3 (P*_cad_::opuD*) | pBK123 with the *opuD* ORF cloned behind the cadmium-inducible promoter at the *Sal*I/*Xma*I sites | This study |
| pML4 (P*_cad_::proP*) | pBK123 with the *proP* ORF cloned behind the cadmium-inducible promoter at the *Sal*I/*Xma*I sites | This study |
| pML5 (P*_cad_::putP*) | pBK123 with the *putP* ORF cloned behind the cadmium-inducible promoter at the *Sal*I/*Xma*I sites | This study |

References

1. P. D. Fey *et al.*, A genetic resource for rapid and comprehensive phenotype screening of nonessential *Staphylococcus aureus* genes. *MBio* **4**, e00537-00512 (2013).

2. B. K. Sharma-Kuinkel *et al.*, The *Staphylococcus aureus* LytSR two-component regulatory system affects biofilm formation. *J Bacteriol* **191**, 4767-4775 (2009).

3. E. Charpentier *et al.*, Novel cassette-based shuttle vector system for gram-positive bacteria. *Appl Environ Microbiol* **70**, 6076-6085 (2004).
