# Supplementary material for: Proline transporters ProT and PutP are required for Staphylococcus aureus infection
Source: PLoS Pathog. 2023 Jan 18;19(1):e1011098. doi: 10.1371/journal.ppat.1011098 (PMC9886301; doi:10.1371/journal.ppat.1011098)
Supplement: S2 Table — (DOCX) [file ppat.1011098.s008.docx]

**S2 Table. Primers used in these studies.**

| **Name** | **Sequence** | **Comments** |
| --- | --- | --- |
| putPup_F | CCGAATTCTATGGTCAAATGGGTTAACTG | Forward primer for *S. aureus* JE2 *putP* upstream region |
| putPup_R | CCGAGCTCCATGATAAACACTCCCTTTATTTAATTTA | Reverse primer for *S. aureus* JE2 *putP* upstream region |
| putPdown_F | CCGAGCTCTAATTCATAAGTCTTAACAAATTAAAAAGGT | Forward primer for *S. aureus* JE2 *putP* downstream region |
| putPdown_R | CCGTCGACTGATAACTATCGTACATTGTTACCA | Reverse primer for *S. aureus* JE2 *putP* downstream region |
| 03660up_dhfR_F | cgtcttcaagaattcgagctTTTAATAAAATCTGGAGAAATAGGAG | Forward primer for *S. aureus* JE2 *proT* upstream region |
| 03660up_dhfR_R | actgtagccgATTCATCATCATGTGAATAATAGAC | Reverse primer for *S. aureus* JE2 *proT* upstream region |
| 03660down_dhfR_F | tgaatatttaaaaTAATATCATATGAGGGATATCCG | Forward primer for *S. aureus* JE2 *proT* downstream region |
| 03660down_dhfR_R | tctagaggatccccgggtacCTACTGGTACATTCAATTCTTG | Reverse primer for *S. aureus* JE2 *proT* downstream region |
| dhfR_F | gatgatgaatCGGCTACAGTGATAACATC | Forward primer for amplifying dhfR ORF |
| dhfR_R | tatgatattaTTTTAAATATTCATGTGATAAATCAGAG | Reverse primer for amplifying dhfR ORF |
| opuDup_erm_F | CCGAGCTCCTTACATTGGAAAACTTTGTCTT | Forward primer for *S. aureus* JE2 *opuD* upstream region |
| opuDup_erm_R | CCGGTACCCATAATTAATTACTCCCTTCAATTCTATA | Reverse primer for *S. aureus* JE2 *opuD* upstream region |
| opuDdown_erm_F | CCGGTACCGATAACTAATAAAGTTTAGTTAAGTATTTTAA | Forward primer for *S. aureus* JE2 *opuD* downstream region |
| opuDdown_erm_R | CCGTCGACCCATCTTTTCCAAACTGACT | Reverse primer for *S. aureus* JE2 *opuD* downstream region |
| erm_opuDF | CCGGTACCAATTGAATGAGACATGCTACACC | Forward primer for amplifying *ermC* |
| erm_opuDR | CCGGTACCAAAACTGGTTTAAGCCGACT | Reverse primer for amplifying *ermC* |
| 03660_pBK123F | GG*CTGCAG*AAGGAGGTCTATTATTCACATGAT | Forward primer for amplifying the JE2 *proT* ORF to clone into pBK123 |
| 03660_pBK123R | CC*GTCGAC*TTAAGCAATATGTTTTTTATCCGC | Reverse primer for amplifying the JE2 *proT* ORF to clone into pBK123 |
| opuC_pBK123F | GG*CTGCAG*TGAGGAAGGAGAAATGATTATGTT | Forward primer for amplifying the JE2 *opuCabcd* ORF to clone into pBK123 |
| opuC_pBK123R | CC*GTCGAC*TTACATATTAATACTTTGCGGCC | Reverse primer for amplifying the JE2 *opuCabcd* ORF to clone into pBK123 |
| opuD_pBK123F | GG*CTGCAG*TATAGAATTGAAGGGAGTAATTAATTATG | Forward primer for amplifying the JE2 *opuD* ORF to clone into pBK123 |
| opuD_pBK123R | CC*GTCGA*CTTAGTTATCTTTTTTCTCTATATTTCTACG | Reverse primer for amplifying the JE2 *opuD* ORF to clone into pBK123 |
| proP_pBK123F | GG*CTGCAG*CAAGAGGGAGTGTATGAATTCA | Forward primer for amplifying the JE2 *proP* ORF to clone into pBK123 |
| proP_pBK123R | CC*GTCGAC*CTCTAATTCTTACGTTCTTTAACCC | Reverse primer for amplifying the JE2 *proP* ORF to clone into pBK123 |
| putP_pBK123F | GG*CTGCAG*TAAAGGGAGTGTTTATCTATGCTT | Forward primer for amplifying the JE2 *putP* ORF to clone into pBK123 |
| putP_pBK123R | CC*GTCGAC*TTATTTTTCTCTAACGATGTCACG | Reverse primer for amplifying the JE2 *putP* ORF to clone into pBK123 |
